# COVID-19 and Surgical Practice in Slovenia: Managing the Crisis in Neurosurgery during the COVID-19 Pandemic

**DOI:** 10.3390/life13102095

**Published:** 2023-10-21

**Authors:** Matic Munda, Tomaz Velnar, Roman Bosnjak, Tilen Zele, Lidija Gradisnik, Peter Spazzapan, Natasa Kos, Nina Kocivnik, Mitja Benedicic, Borut Prestor

**Affiliations:** 1 Department of Neurosurgery, University Medical Centre Ljubljana, 1000 Ljubljana, Slovenia; matic.munda@gmail.com (M.M.); roman.bosnjak@kclj.si (R.B.); tilen.zele@kclj.si (T.Z.); spazzapanpeter@yahoo.it (P.S.); mitja.benedicic@kclj.si (M.B.); borut.prestor@kclj.si (B.P.); 2 AMEU-ECM Maribor, 2000 Maribor, Slovenia; natasa.kos@kclj.si; 3 Institute of Biomedical Sciences, Medical Faculty Maribor, 2000 Maribor, Slovenia; lidija.gradisnik@um.si; 4 Department of Rehabilitation, University Medical Centre Ljubljana, 1000 Ljubljana, Slovenia; 5 Faculty of Pharmacy, University of Ljubljana, 1000 Ljubljana, Slovenia; kocivnik.nina@gmail.com

**Keywords:** COVID-19, pandemic, neurosurgery, Slovenia, anti-COVID-19 measures

## Abstract

Worldwide, the novel coronavirus disease 2019 (COVID-19) has become a significant threat to global health. Worldwide, COVID-19 has affected the health service also in Slovenia. During this time, neurosurgery is facing difficulties in its service, both in emergency and elective surgeries. In the article, we describe the anti-COVID-19 measures taken at our neurosurgical department in a medical centre in Ljubljana, Slovenia, and analysed and compared the number of emergency and elective neurosurgical procedures during the time of the pandemic.

## 1. Introduction

COVID-19 is a novel coronavirus disease caused by severe acute respiratory syndrome coronavirus-2 (SARS-CoV-2) [1,2]. In December 2019, the disease was first discovered in Wuhan in China. A few months later, a pandemic was officially declared by the World Health Organisation (WHO) on 11 March 2020 [3,4].

Severe and unique biological features, specific clinical symptoms, particular blood test results and imaging findings designate the disease. Because of its high contagiousness and rapid spread, the virus has been recognised as a major threat to global health [2,5,6]. At the moment of writing this article, in August 2023, the number of infected patients has exceeded 600 million in almost every country in the world. The actual number of infections is most likely even higher. The death toll that was caused by COVID-19 is estimated at a value higher than 6.5 million [1,7]. 

In most people, signs of infection appear after an incubation period of 1 to 14 days, and respiratory distress and pneumonia develop within 10 days of onset. All age groups are susceptible to the infection, but the clinical picture depends on the patient age. Most young people have mild or even asymptomatic symptoms, while people over 60 years of age are more prone to develop severe respiratory illness leading to hospitalisation or, in severe cases, death [8]. The WHO reports that in 80%, the infections are mild to moderate, and 13.1% of infected patients develop a severe form of disease. In 6.1% of those infected, intensive treatment is required [8,9].

Risk factors for developing a more severe course include an age over 60 years and concomitant chronic diseases, such as hypertension, obesity, diabetes, cardiovascular disease, immunodeficiency, underlying lung disease and certain physiological states, such as pregnancy [9,10]. Some patients may develop long-lasting effects of infection after infection with SARS-CoV-2, referred to as long COVID. This is defined by signs and symptoms that develop during or after infection that are consistent with COVID-19, last longer than 12 weeks and cannot be explained with another diagnosis. They are usually manifested with a cluster of symptoms that can often overlap, fluctuate and change over time, and can affect any system in the body [10,11,12].

## 2. The Pandemic of COVID-19 and Medical Services

The pandemic of COVID-19 caused problems and complications in almost every healthcare system. European countries, which were severely affected and experienced almost similar troubles, had to make necessary adjustments in order to provide the required care to COVID-19 patients. On the other hand, they had to ensure the functioning of the health system as normally as possible [3]. Recommendations were issued by the European Union for its member states on how to approach the pandemic. Unquestionably, there were differences between European countries in their approach to the COVID-19 crisis and health policies [4,13]. Many hospital protocols were established and protective measures were taken de novo to manage the immense inflow of COVID-19 patients and to maintain the normal operation of medical services. Redistribution and protection of medical staff was needed, as well as the equipment and supplies. It was necessary to create management protocols, establish reserve hospitals and rearrange and regulate the internal routes and adjust operating theatres for sick patients. Strict control of elective and/or emergency admissions, prevention of mixing of cases and medical staff, improvement in surgical and treatment processes, allocation of designated areas for admission and surgery of COVID-19 patients and strict ward management were put into practise [3,13,14,15]. With the help of telemedicine, the conventional outpatient service was transformed into an outpatient service on distance. Preventive measures and programmes came at a standstill or were slowed down. Elective surgeries were postponed or stopped. In all this chaos, some patients infected with COVID-19 had to undertake vital surgical procedures, whereas others developed the symptoms in a few days after elective surgeries. These all had to be taken care of. In spite of public health actions worldwide aimed at limiting the disease and delaying its spread, several countries were facing a health crisis [3,13,15,16].

As in other European countries, COVID-19 has greatly affected the health services in Slovenia. Slovenia is located in Central Europe and its population count is two million. The neurosurgical centres are located in university hospitals. There are two of them. One neurosurgical centre is in the capital city of Ljubljana, which has 280,000 residents. The second largest neurosurgical centre is in Maribor. With the population of 95,000, this is also the second largest city in Slovenia. Both centres deal with all types of neurosurgical pathology. The neurosurgical centre in Ljubljana covers about two-thirds of the country’s population and the neurosurgical centre in Maribor covers one-third. The initial infections with COVID-19 in Slovenia were reported almost at the same time as infections were detected in other countries in Central Europe. Because of a prompt virus spread, it was practically not possible to arrange extensive preparations for the pandemic and to adjust the health system to a situation that was never encountered before. At the state level, official anti-virus measures were initiated a few days after the confirmation of the first case, on 4 March 2020. A week later, the epidemic was officially declared [14].

Among all specialties, neurosurgery also faced difficulties. This was evident not only in regular, elective operations, but particularly in emergency situations. Therefore, the treatment of neurosurgical patients has become more difficult. Most urgent neurosurgical patients require immediate consultation and intervention to achieve an optimal treatment outcome. Therefore, promptness and swiftness of treatment are crucial [15,16,17,18,19]. The initial factor here includes mode and time of transport. The time to neurosurgical treatment and the distance of transport to our hospital with adequate neurosurgical care significantly predict mortality and outcome. Approximately 10% of patients with an average transport time of just over 4 hours experience a drop in the Glasgow Coma Scale during transport [19,20]. Those delayed by more than 4 hours have a higher mortality rate than those admitted directly to the neurosurgical service. Distance to the neurosurgical centre is one of the main aspects for delay in the treatment of neurosurgical emergencies. The second problem is the possible COVID-19 infection [18,21,22,23]. The usual transport time to our centre varies between 1 and 4 hours, which is subject to the type of transport, namely helicopter or road transport. All urgent patients were first subjected to a viral test, and even in the best-case scenario, the rapid antigen test takes 1 to 2 hours to perform, prolonging neurosurgical management. The polymerase chain reaction (PCR) test, which has a higher sensitivity, takes even longer to perform. It is therefore not suitable for neurosurgical emergencies. In addition, emergency patients were treated according to special anaesthesiological and surgical procedures. Protective equipment, such as face masks, eye protection, eye shields, gowns and special gloves were worn. The influx of personnel into and out of the operating theatre was kept to a minimum. Special care was taken with equipment and postoperative care. All these challenges affected patient care and outcomes, and there were significant costs to the health system.

The preventive measures were also taken at the state level, including state lockdowns, mandatory masks, hand disinfection, testing and strict border crossing rules, and a number of protocols in hospitals and departments were put in place to limit the spread of the virus within health centres and hospitals. The implemented measures encompassed adjustments in outpatient services, strict rules in patient admissions, postponement of elective surgeries, additional separate rooms at the department and operating rooms for high-risk and COVID-19-positive patients, reductions in the surgical programme, constant use of protective equipment, expansion of telephone counselling services and reallocations of staff to COVID-19-related positions [15]. The use of outpatient telemedicine and telephone consultations increased, and adjustments and improvements were made in surgical practise and the treatment process. In addition, strict ward management, the designation of high-risk areas for the accommodation and operation of COVID-19 patients and the prevention of patient and health worker mixing became more important than ever [15,16].

This article describes the impact COVID-19 had on the treatment of neurosurgical patients in the neurosurgical department of Ljubljana.

## 3. The Management Protocol

In the period before the COVID-19 epidemic, the patients with elective neurosurgical pathology were admitted directly to the neurosurgical department at the university medical centre in Ljubljana. In urgent cases, they were transferred from the emergency rooms. They underwent a thorough clinical examination, neuroimaging and routine tests. The elective patients were operated on only in two elective operating theatres and the urgent patients in one emergency operating room. The latter was strictly reserved only for urgent cases. From March 2018 to March 2019, before the pandemic, 1311 elective patients were addressed. A total of 376 brain tumour patients underwent surgery, and there were 43 vascular procedures, 46 endoscopic endonasal surgeries, 87 hydrocephalus surgeries (excluding external ventricular drainage), 612 spinal surgeries, 72 procedures in functional neurosurgery and 321 miscellaneous emergencies, together with traumatic brain injuries (Table 1).

From March 2020 to March 2021, when the epidemic was declared in Slovenia, we surgically treated 1213 patients. The pathology was diverse: 314 brain tumours, 39 vascular procedures, 65 hydrocephalus surgeries, 362 spinal surgeries and 52 procedures in functional neurosurgery. Other patients suffered from diverse types of vital pathologies, again including patients with severe traumatic brain injury (Table 1). We have established since then a dedicated emergency neurosurgical operating theatre, operating on COVID-19-positive patients, strategically positioned in the emergency zone, in proximity to all major emergency patient admission rooms.

## 4. The Initial Phase of the COVID-19 Epidemic

During the pandemic, the first step in neurosurgical patient referral was screening and safety assessment. In the early stage of the epidemic, when the number of positive COVID-19 patients was still low and anti-virus measures were taken (e.g., lockdown), admission of all urgent patients was restricted to the emergency department. Urgent patients were treated immediately and simultaneously screened for potential risk of infection with COVID-19, first with a nasopharyngeal swab for the rapid antigen test (RAT) and then with a second swab for the reverse transcriptase polymerase chain reaction test (RT-PCR). In contrast, elective patients and patients who did not need to be transferred urgently from elsewhere were provisionally located in the transition zones or specially equipped waiting areas, where the RT-PCR was carried out prior to surgery. The patients were tested for a possible risk of infection with COVID-19 using the RT-PCR test. After the results of testing were available, they were transferred to a regular department to avoid possible spread of the virus. In addition, all persons accompanying the patients were asked to fill out the questionnaire and to observe strict protective measures upon admission. The emergency area was reconfigured to include a special isolation area where urgent patients were admitted for examination. All emergency, support and critical care equipment was available. This zone was isolated and no connection to other (secure) parts of the hospital were possible. The primary screening procedure involved beside body temperature measurements and also a COVID-19 screening questionnaire. This also applied to patients’ relatives and caregivers, particularly for patients who were not conscious, and to the strict use of protective measures, including face masks and hand disinfection. Preoperative procedures, imaging and surgical and early postoperative care were accomplished with the protective procedures in COVID-19-positive patients and in those where the nature of their disease necessitated urgent surgery. In negative patients, treatment was normal. 

## 5. The Final Phase of the COVID-19 Epidemic

In the late phases of the epidemic, the number of infected patients rose. Therefore, the previously described scheme had to be modified. To isolate the neurosurgical patients treated at our centre, all areas and patients were separated into three groups: (I) red (danger zone for urgent patients), (II) grey (transitional, waiting zone for elective patients) and (III) green (safe zone for elective patients). The red zone was designated for high-risk patients. Preventive and protective measures were respected during their management. For patients who needed immediate surgery, the RAT was performed. The RT-PCR was performed during surgery to place these patients in appropriate postoperative hospital areas. For surgery of these urgent patients, full personal protective equipment was used. The second, grey group involved all elective and non-urgent patients, who were treated at the neurosurgery department for elective pathology. This group included the vaccinated or the pre-tested patients (before admission), who were placed in the grey areas to assess their COVID-19 status using the RT-PCR. This represented the ultimate test before a patient was moved to the green zone for elective surgery. If the virus was confirmed negative, they were relocated to the green zones. Any transferral between these hospital and departmental areas was limited, including equipment, supplies and staff. The green zone was the safe zone. Only elective patients that were COVID-19-negative were accommodated here.

This protocol was implemented for all emergency patients. All admitted patients were categorised according to the level of emergency for intervention. Those with life-threatening emergencies were operated on immediately, irrespective of COVID-19 status. This group involved a small number of patients that suffered from serious neurosurgical emergencies, those who were being treated in other departments and those whose COVID-19 status was certainly negative at the time their health condition had worsened. Every week, all patients had their COVID-19 swabs taken regularly as a control. The inclusion criteria for emergency surgery included four categories: (I) all paediatric and adult patients with features of increased intracranial pressure, such as stroke, abscess, tumour, cerebral oedema, epidural or subdural haematoma and worsening hydrocephalus; (II) all traumatic cases requiring observation or emergency surgery; (III) all spinal compression myelopathies (both traumatic and non-traumatic); and (IV) vascular emergencies—ruptured arteriovenous malformations, ruptured intracranial aneurysms and intracerebral haematomas. The urgent cases were categorised according to the complexity of the case, availability of surgical instruments, skills of the anaesthesiological team and accessibility of postoperative accommodation. Patients with neurosurgical emergencies, who could be treated with the existing facilities, were operated on in the designated operating theatres according to the causative pathology. All protective measures were applied. These patients were postoperatively treated in intensive care units in the designated red zones until they were fit for discharge.

Patients with no known exposure to COVID-19 and COVID-19-negative patients, i.e., elective patients, semi-urgent patients and patients without signs and symptoms of acute respiratory infections with normal chest X-ray and negative RT-PCR COVID-19 tests, were considered low-risk patients. The low-risk patients were operated on in a standard (pre-COVID-19) neurosurgical setting. After the operation, they recovered in green areas, both in the intensive care units and at the department.

**Figure 1:**
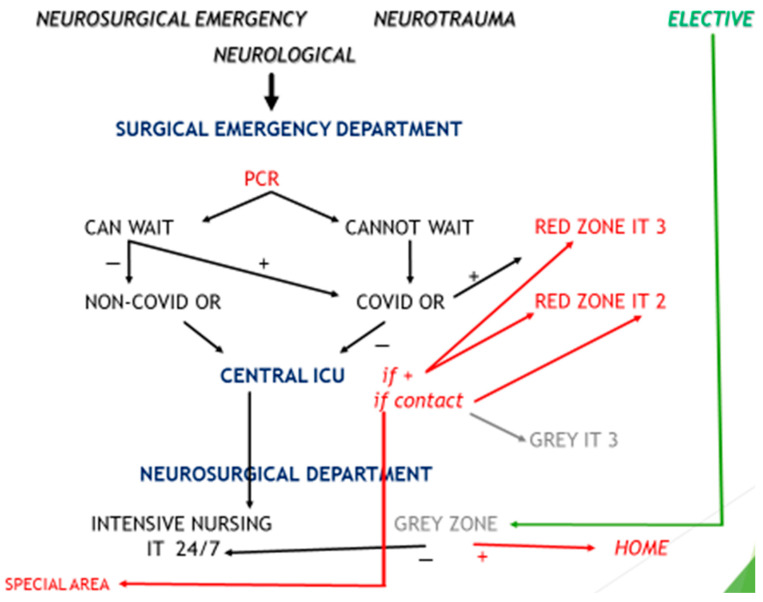
A graphic representation of neurosurgical patient management during the pandemic. All neurosurgical patients were first addressed at the surgical general emergency department. Primary screening for COVID-19 followed and the patients were triaged—separated into two groups: (I) to those who could not wait and (II) to those who could wait. (I) The patients from group one represented the urgent group and they were operated on immediately in the COVID-19 operation theatre. During surgery, the RT-PCR test was repeated. If the result was positive, these patients were transferred into the red zones, including the COVID-19 ICUs. When patients contracted COVID-19 in the course of the hospitalisation, they were transported to the red areas or discharged home, depending on the clinical condition. The patients who were in contact with COVID-19 and were not confirmed positive were observed and treated in grey areas. (II) Non-urgent patients were managed according to the RT-PCR test. If confirmed negative, they were treated in green areas. If confirmed positive, they were treated in red areas. Non-urgent patients were examined in grey areas first and waited there for the RT-PCR test. Positive patients were sent home. Negative patients were hospitalised in green areas.

The schematic representation of the flow of neurosurgical patients at the university medical centre in Ljubljana is summarised in Figure 1. 

## 6. Personal Protective Measures

During the admission into the emergency department, all patients were examined by a group of physicians: an anaesthesiologist, a traumatologist and a neurosurgeon. All staff who came into contact with patients adhered to protective measures. In addition to protective clothing and gloves, N95 or FFP3 masks and face shields were worn. Personal protective equipment was provided for support staff. The high-risk patients were operated on by a neurosurgeon and a neurosurgical assistant. All neurosurgical staff wore face shields and N95 or FFP3 masks in addition to the standard surgical gown and protective clothing. The anaesthesia and support staff in the operating room applied similar protective procedures. The anaesthetist and the assistant remained in the operating theatre when intubation was in progress. Other members of the staff waited outside until the procedure was accomplished. As soon as the patient was attached to the ventilator, they entered the operating theatre. During the craniotomy and bone drilling with copious irrigation, additional transparent face shields or ski goggles were worn. The electrosurgical knives were connected to suction devices when possible to eliminate contaminated smoke from electrocoagulation. Movement was restricted to one entrance and exit and the floor was disinfected with chlorhexidine solution and alcohol. Additionally, a disinfectant-soaked mat was placed at the entrances and exits to limit the possible spread of the virus on footwear. The outdoor support workers wore face shields in addition to their three-layer surgical mask and protective clothing. The air conditioning was turned off for the duration of the surgery and the operating theatre was thoroughly disinfected afterwards. The same precautions were taken for COVD-19-positive patients in the intensive care unit or in the neurosurgical department. The RT-PCR was performed twice a week on a regular basis or as needed during the recovery period. In addition, patients were discharged as soon as possible to create capacity for further cases.

In the grey areas, protective equipment comprised of disposable clothing and N95 or FFP3 masks with goggles or face shields. Dressing and disinfecting were mandatory at the entry. In green areas, medical service was normal. The protective measures included goggles and three-layer surgical masks. Twice a week, nasal swabs for RT-PCR were taken for control. When possible, patients were discharged home earlier, usually forgoing the normal postoperative rehabilitation, which usually takes a few days longer, at least in our country.

## 7. Methods

The number of elective and emergency surgical procedures was analysed in the period before and during the pandemic. We defined emergency as any patient that required urgent surgery within a few hours due to a life-threatening condition, and elective surgery as any case that was planned in advance and operated on during regular working time. 

### Patient Analysis

For the analysis, the interventions were divided into subgroups: (I) spinal pathology (degenerative diseases of the spine, lesions of the spine), (II) lesions of the brain (any supra- or infratentorial brain tumours, abscesses, cysts or similar pathologies), (III) ventriculoperitoneal drainage, (IV) cranioplasty, (V) procedures in functional neurosurgery (spinal cord stimulation, battery replacement, vagus nerve stimulation, deep brain stimulation), (VI) vascular pathology (aneurysms, arteriovenous malformations…) and (VII) endonasal endoscopy (pituitary adenoma, Rathke’s cyst, clivus chordoma, craniopharyngioma). Any procedure that required immediate surgical intervention was considered vital or emergency surgery. This included any form of acute cerebral haemorrhage (subdural, epidural or intracerebral), placement of external ventricular drainage or intracranial pressure monitoring, decompressive craniectomy and removal of a chronic subdural haematoma, or vital spinal disease.

## 8. Results

We analysed patient numbers and pathology 1 year before the announcement of the COVID-19 epidemic (from March 2018 to March 2019). During this period, 1632 patients were operated on at our department. Of these, 1311 patients comprised elective cases, among which were a total of 612 spinal pathologies, 376 brain lesions, 87 elective ventriculoperitoneal drains for the treatment of hydrocephalus and 32 cranioplasties, and 72 functional, 43 vascular, 46 endonasal endoscopic and 43 other procedures. We performed 321 emergency procedures.

**Figure 2:**
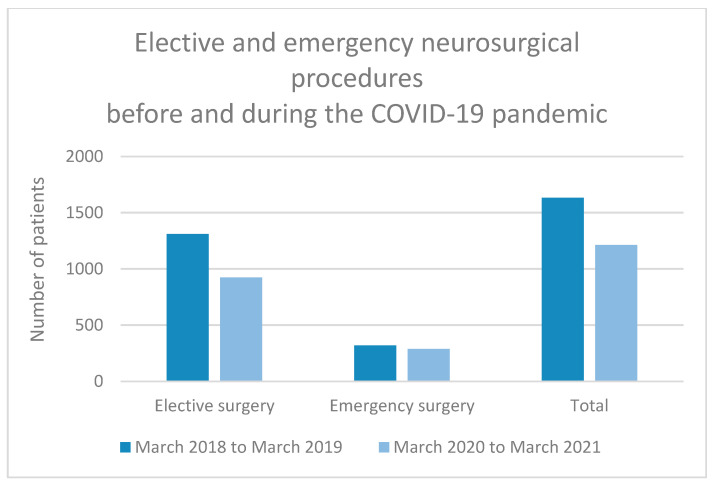
Elective and emergency operations before and after the COVID-19 epidemic.

**Figure 3:**
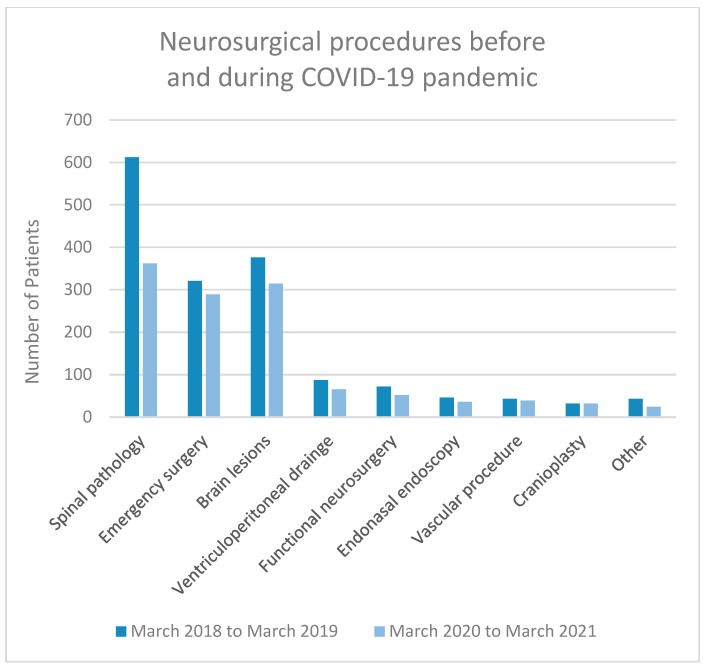
The subgroups of neurosurgical procedures before and during the COVID-19 epidemic.

One year after the COVID-19 epidemic was declared, from March 2020 to March 2021, we had surgically treated 1213 patients. Elective procedures included 924 patients. There were 362 spine surgeries, 314 brain lesions, 65 ventriculoperitoneal drains and 32 cranioplasties, and 52 functional, 39 vascular, 36 endoscopic endonasal and 24 other procedures. Emergencies occurred in 289 cases (Figure 2 and Figure 3).

A decrease of 25.7% (*n* = 419) was recorded in all neurosurgical procedures in 1 year following the declaration of COVID-19 compared to 1 year before. The decrease in elective procedures was 29.5% (*n* = 387) and was mainly due to spine surgeries (40.9% decrease or 250 cases), functional neurosurgical procedures (27.8% decrease or 20 cases), endonasal endoscopy procedures (21.7% decrease or 10 cases) and brain lesions (16.5% decrease or 62 cases). The decrease in emergency procedures was also noted and amounted to 10% (*n* = 32) (Table 1). We performed a Chi-Squared test to analyse the association between a type of neurosurgical procedure (elective or emergency) and the time period (before or during COVID-19 epidemic), both of which are categorical variables. The Chi-Squared test of independence revealed a statistically significant association between a type of procedure and the period before or during the COVID-19 epidemic (χ^2^ = 7.14, df = 1, *p* < 0.01). This indicates that there is a significant association between the type of neurosurgical procedure performed and the period when the surgery was performed and was influenced by the COVID-19 epidemic.

The patients treated at the university hospital in Ljubljana during the pandemic were exclusively Slovenian citizens. Sometimes we also accept neurosurgical patients from other countries, these being mainly tourists visiting Slovenia. In the era of COVID-19, the borders were closed and therefore the flow of tourists and visitors was reduced or even stopped. Also, within the country, daily migrations were stopped to contain the spread of the virus.

## 9. Discussion

During the epidemic, the neurosurgical practise had to be adapted and organised according to new rules [14,24]. In spite of the problems, which the virus brought to the health systems worldwide, it was essential to take into account that regular medicine had to work uninterruptedly and alongside with the treatment of COVID-19 patients [24,25]. As expected, the influx of patients into medical facilities did not decrease during the epidemic, but rather rose. The COVID-19 infections were mixed with everyday medical problems that needed to be treated appropriately and professionally as well. This was especially significant for neurosurgery, where admitted emergencies must be treated swiftly to avoid causing additional harm to the nervous system [26,27]. Therefore, neurosurgical practise had to be organised according to new rules. 

Our neurosurgery department is concerned with all types of neurosurgical pathology from the whole country. The influx of patients was constant during the epidemic and it was therefore essential to divide the patients according to the priority of treatment. We introduced a triage system at the outpatient clinic level to minimise patient intake and adapt to the new situation. We would like to stress that all urgent patients were treated immediately and without postponement.

All surgical COVID-19 patients were treated in special COVID-19 zones or areas. Because of our rigorous screening policy and case selection, the safety of our patients during the epidemic increased. Only two COVID-19 infiltration cases were recorded. Luckily, these patients were detected as positive before their scheduled surgery and were discharged home. One patient, who was positive after surgery, was transferred to the COVID-19 treatment facility.

Comparing the number of neurosurgical procedures 1 year before and 1 year after the epidemic of COVID-19 reveals that there was approximately a 25% decrease, especially in elective cases. The decrease in the number of patients was most pronounced in spinal pathology and functional neurosurgery. These types of surgery were the least urgent, as the pathology was not malignant and there was no tendency towards neurological deterioration. As for spinal pathology, the decrease was mainly due to degenerative spinal disease with pain syndromes. Patients with associated neurological deficits or spinal lesions were treated promptly and operated on as quickly as possible. The number of procedures in other neurosurgical areas was comparable. The number of brain and spine lesions decreased slightly, which could be coincidental or due to the fact that for a time during the peak of the epidemic, patients without acute clinical manifestations did not manage to reach adequate health services and did not receive an appropriate diagnosis in time. During the pandemic, the reduction in elective procedures was not as extreme as we had anticipated. One of the explanations for this was the effective COVID-19 screening protocol. Very few operations were postponed because a patient was SARS-CoV-2-positive, because if the patient tested positive on admission, they were transferred and sent home and a replacement patient was brought in for the operation. An additional reason could be that during the quieter times of the epidemic, when medical staff returned to their original jobs, more operations were accomplished to catch up.

In other SARS-CoV-2-positive patients, conservative treatment was considered when possible or the patient was deferred for surgery until the infectious period had elapsed. These measures optimised the management of neurosurgical emergencies in a way that patients were not deprived of immediate neurosurgical intervention. We confirmed this using the number of emergency interventions 2 years before and after the COVID-19 epidemic. This compares with 321 emergency surgeries performed from March 2018 to March 2019 and 289 surgeries from March 2020 to March 2021. Despite the constraints and the epidemiological situation, a significant quantitative decrease in urgent neurosurgical services was not recorded.

It is very important to treat patients carefully and correctly in operating theatres to limit the possible spread of the virus [28]. Rapid antigen screening and zoning of hospitals were practical measures that have proved to be very successful [28,29,30]. All nursing staff and attending physicians had to observe the protective measures, including appropriate masks, face shields, proper clothing and gloves. Half- or full-face masks were an alternative to an N95 or FFP3 mask. If available, powered respirators can also be used, which offer good effectiveness and better protection [31,32,33]. Negative pressure suction rooms are preferred for performing surgical procedures in these situations [22,34]. 

In addition to rapid sequence intubation, which prevented dispersion of aerosols, special measures for anaesthesiological procedures involved emptying the operating theatre during intubation. High-risk anaesthetic procedures included intubation and extubation, face mask ventilation, aspiration of airway secretions, high-flow nasal oxygen and cardiopulmonary resuscitation [35,36,37,38]. Low-risk interventions included external ventricular drainage and placement of a lumbar drain. Only necessary equipment was kept in the operating theatre, and disposable equipment was used whenever possible. Extubation was performed on the operating table as this lowers the possibility of cross-infection. Sufficient time was permitted for appropriate disinfection of the operating theatre. Besides usual precautions of minimal drilling and extensive irrigation, we avoided endoscopic transnasal surgery (for pituitary tumours) unless there were urgent indications (apoplexy, loss of vision). In addition, a clear distribution of roles, a disinfection and ventilation plan and mutual monitoring of all staff for possible contamination while reducing the number of people in the operation theatre were beneficial [22,31,37,38,39].

The new triage systems, which were established in the outpatient clinic, helped to optimise patient admission. This new triage system classified patients according to pathology and the need for interventions and they were treated according to their needs and operated on if necessary. Procedures that could buy time, such as non-urgent spinal surgery and some specialised procedures including awake surgery, were deferred. When possible, conservative treatment, at least for a period of time, was recommended [29]. Elective surgeries, including vascular, tumour, hydrocephalus and semi-urgent elective spine surgeries, were almost normal. During the epidemic, the waiting time for these operations did not change [36,37,38,39,40,41].

After the end of the epidemic, the volume of surgical procedures has returned to previous levels. Moreover, since fewer patients were treated during the period of COVID-19, we had to catch up on the delayed cases. These were of course not the urgent patients, but patients with elective pathology. They were all operated on after the epidemic was over. Unfortunately, neurological sequelae of the disease occurred in some of them, as was also seen in other surgical specialties. After the COVID-19 pandemic was officially over, we started operating and working normally. 

## 10. Conclusions

COVID-19 has exerted a remarkable influence on the healthcare system in Slovenia. Despite the troubles, the medical society tried to ensure that medical services were running as smoothly as possible, not only for neurosurgical patients, but also for those in need of other medical specialties. The treatment practice was continuously changing and adapting according to the course of the epidemic. By adhering to the stringent testing and triage system, segregating patients and maintaining protective measures for both patients and staff, we have succeeded to assure continuous neurosurgical service in the course of the COVID-19 epidemic. In the future, we will nevertheless need improved strategies to confront a new potential epidemic without jeopardising the normal healthcare system, especially for life-saving interventions in critical specialties such as neurosurgery.

## Figures and Tables

**Table 1:** The presentation of neurosurgical procedures in the period before and after the COVID-19 epidemic was announced.

Type of Neurosurgical Procedure	Number of Cases		Decrease in Number of Cases
	March 2018 to March 2019	March 2020 to March 2021	
Spinal pathology	612	362	250 (40.9%)
Emergency surgery	321	289	32 (10%)
Brain lesions	376	314	62 (16.5%)
Ventriculoperitoneal drainage	87	65	22 (25.3%)
Functional neurosurgery	72	52	20 (27.8%)
Endonasal endoscopy	46	36	10 (21.7%)
Vascular procedure	43	39	4 (9.3%)
Cranioplasty	32	32	/
Other	43	24	19 (44.2%)
Elective surgery	1311	924	387 (29.5%)
Emergency surgery	321	289	32 (10%)
Total	1632	1213	419 (25.7%)

## Data Availability

Not applicable.

## References

[1] Haidar M.A., Shakkour Z., Reslan M.A., Al-Haj N., Chamoun P., Habashy K., Kaafarani H., Shahjouei S., Farran S.H., Shaito A. (2022). SARS-CoV-2 involvement in central nervous system tissue damage. Neural Regen. Res..

[2] Peacock W.F., Soto-Ruiz K.M., House S.L., Cannon C.M., Headden G., Tiffany B., Motov S., Merchant-Borna K., Chang A.M., Pearson C. (2022). Utility of COVID-19 antigen testing in the emergency department. J. Am. Coll. Emerg. Physicians Open.

[3] Doglietto F., Vezzoli M., Gheza F., Lussardi G.L., Domenicucci M., Vecchiarelli L., Zanin L., Saraceno G., Signorini L., Panciani P.P. (2020). Factors Associated With Surgical Mortality and Complications Among Patients With and Without Coronavirus Disease 2019 (COVID-19) in Italy. JAMA Surg..

[4] Clavien P.A., Barkun J., de Oliveira M.L., Vauthey J.N., Dindo D., Schulick R.D., De Santibañes E., Pekolj J., Slankamenac K., Bassi C. (2009). The Clavien-Dindo classification of surgical complications: Five-year experience. Ann. Surg..

[5] Chams N., Chams S., Badran R., Shams A., Araji A., Raad M., Mukhopadhyay S., Stroberg E., Duval E.J., Barton L.M. (2020). COVID-19: A Multidisciplinary Review. Front. Public Health.

[6] Murta V., Villarreal A., Ramos A.J. (2020). Severe Acute Respiratory Syndrome Coronavirus 2 Impact on the Central Nervous System: Are Astrocytes and Microglia Main Players or Merely Bystanders?. ASN Neuro..

[7] World Health Organization (2022). WHO Coronavirus (COVID-19) Dashboard (Internet). https://covid19.who.int.

[8] Hu B., Guo H., Zhou P., Shi Z.L. (2021). Characteristics of SARS-CoV-2 and COVID-19. Nat. Rev. Microbiol..

[9] Adil M.T., Rahman R., Whitelaw D., Jain V., Al-Taan O., Rashid F., Munasinghe A., Jambulingam P. (2021). SARS-CoV-2 and the pandemic of COVID-19. Postgrad. Med. J..

[10] Saini L., Krishna D., Tiwari S., Goyal J.P., Kumar P., Khera D., Choudhary B., Didel S., Gadepalli R., Singh K. (2022). Post-COVID-19 Immune-Mediated Neurological Complications in Children: An Ambispective Study. Pediatr. Neurol..

[11] Pires R.E., Reis I.G.N., Waldolato G.S., Pires D.D., Bidolegui F., Giordano V. (2022). What Do We Need to Know About Musculoskeletal Manifestations of COVID-19?: A Systematic Review. JBJS Rev..

[12] Russell D., Spence N.J., Chase J.D., Schwartz T., Tumminello C.M., Bouldin E. (2022). Support amid uncertainty: Long COVID illness experiences and the role of online communities. SSM Qual. Res. Health.

[13] Suleyman G., Fadel R.A., Malette K.M., Hammond C., Abdulla H., Entz A., Demertzis Z., Hanna Z., Failla A., Dagher C. (2020). Clinical Characteristics and Morbidity Associated With Coronavirus Disease 2019 in a Series of Patients in Metropolitan Detroit. JAMA Netw. Open.

[14] Leira E.C., Russman A.N., Biller J., Brown D.L., Bushnell C.D., Caso V., Chamorro A., Creutzfeldt C.J., Cruz-Flores S., Elkind M.S. (2020). Preserving stroke care during the COVID-19 pandemic: Potential issues and solutions. Neurology.

[15] Arteaga A.S., Aguilar L.T., González J.T., Boza A.S., Muñoz-Cruzado V.D., Ciuró F.P., Ruíz J.P. (2021). Impact of frailty in surgical emergencies. A comparison of four frailty scales. Eur. J. Trauma Emerg. Surg..

[16] Rockwood K., Song X., MacKnight C., Bergman H., Hogan D.B., McDowell I., Mitnitski A. (2005). A global clinical measure of fitness and frailty in elderly people. CMAJ.

[17] Burke J.F., Chan A.K., Mummaneni V., Chou D., Lobo E.P., Berger M.S., Theodosopoulos P.V., Mummaneni P.V. (2020). “Letter: The Coronavirus Disease 2019 Global Pandemic: A Neurosurgical Treatment Algorithm. Neurosurgery.

[18] Vlada Republike Slovenije (2020). Slovenija Razglasila Epidemijo Novega Koronavirusa. https://www.gov.si/novice/2020-03-12-slovenija-razglasila-epidemijo-novega-koronavirusa/.

[19] American College of Surgeons (2020). COVID-19: Recommendations for Management of Elective Surgical Procedures. https://www.facs.org/COVID-19/clinical-guidance/elective-surgery.

[20] Olson S., Honeybul S., Rosenfeld J.V. (2021). Considering Futility of Care Decisions in Neurosurgical Practice. World Neurosurg..

[21] Katayama Y., Kiyohara K., Kitamura T., Hayashida S., Shimazu T. (2020). Influence of the COVID-19 pandemic on an emergency medical service system: A population-based, descriptive study in Osaka, Japan. Acute Med. Surg..

[22] Gulen M., Satar S., Acehan S., Bozkurt M., Aslanturkiyeli E.F., Sevdimbas S., Esen C.I., Balcik M., Uzucek M.D., Sahin G.K. (2022). Have the Diagnoses of Patients Transported by Ambulances Changed in the Early Stage of the COVID-19 Pandemic?. Prehosp. Disaster Med..

[23] NIJZ Koronavirus. https://www.nijz.si/sl/koronavirus-2019-ncov.

[24] Jaswaney R., Davis A., Cadigan R.J., Waltz M., Brassfield E.R., Forcier B., Joyner B.L. (2022). Hospital Policies During COVID-19: An Analysis of Visitor Restrictions. J. Public Health Manag. Pract..

[25] Nejadghaderi S.A., Saghazadeh A., Rezaei N. (2022). Health Care Policies and COVID-19 Prevalence: Is There Any Association?. Int. J. Health Serv..

[26] Agyemang K., Rose A., Baig S., Al Salloum L., Osman A.A., Steckler F., Barrett C. (2021). Neurosurgery in octogenarians during the COVID-19 pandemic: Results from a tertiary care trauma centre. Interdiscip. Neurosurg..

[27] Sander C., Dercks N.V., Fehrenbach M.K., Wende T., Stehr S., Winkler D., Meixensberger J., Arlt F. (2021). Neurosurgical Care during the COVID-19 Pandemic in Central Germany: A Retrospective Single Center Study of the Second Wave. Int. J. Environ. Res. Public Health.

[28] Ozoner B., Gungor A., Hasanov T., Toktas Z.O., Kilic T. (2020). Neurosurgical Practice During Coronavirus Disease 2019 (COVID-19) Pandemic. World Neurosurg..

[29] Panciani P.P., Saraceno G., Zanin L., Renisi G., Signorini L., Fontanella M.M. (2020). Letter: COVID-19 Infection Affects Surgical Outcome of Chronic Subdural Hematoma. Neurosurgery.

[30] Ashkan K., Jung J., Velicu A.M., Raslan A., Faruque M., Kulkarni P., Bleil C., Hasegawa H., Kailaya-Vasan A., Maratos E. (2021). Neurosurgery and coronavirus: Impact and challenges-lessons learnt from the first wave of a global pandemic. Acta Neurochir..

[31] Arimappamagan A., Vilanilam G., Pandey P. (2021). Is Elective Neurosurgery Justified During COVID-19 Pandemic?. Neurol. India.

[32] Dannhoff G., Cebula H., Chibbaro S., Ganau M., Todeschi J., Mallereau C.H., Pottecher J., Proust F., Ollivier I. (2021). Investigating the real impact of COVID-19 pandemic on the daily neurosurgical practice?. Neurochirurgie.

[33] Malhotra N., Joshi M., Datta R., Bajwa S.J., Mehdiratta L. (2020). Indian Society of Anaesthesiologists Advisory and Position Statement regarding COVID-19. Indian J. Anaesth..

[34] Deora H., Dange P., Patel K., Shashidhar A., Tyagi G., Pruthi N., Arivazhagan A., Shukla D., Dwarakanath S. (2021). Management of Neurosurgical Cases in a Tertiary Care Referral Hospital During the COVID-19 Pandemic: Lessons from a Middle-Income Country. World Neurosurg..

[35] Agarwal N., Raheja A., Suri A. (2021). Guidelines for Preoperative Testing for Neurosurgery in Coronavirus Disease 2019 (COVID-19) Era: Indian Viewpoint Amidst Global Practice. World Neurosurg..

[36] Sharma A.K., Gandhoke C.S., Nayak N. (2020). Effect of coronavirus disease 2019 pandemic on case volume, spectrum, and perioperative coronavirus disease 2019 incidence in neurosurgical patients: An experience at a tertiary care center in India. Surg. Neurol. Int..

[37] Shao C.C., McLeod M.C., Gleason L., Marques I.C.D.S., Chu D.I., Gunnells D. (2021). Effect of COVID-19 Pandemic Restructuring on Surgical Volume and Outcomes of Non-COVID Patients Undergoing Surgery. Am. Surg..

[38] Velnar T., Bosnjak R. (2022). Management of neurosurgical patients during coronavirus disease 2019 pandemics: The Ljubljana, Slovenia experience. World J. Clin. Cases.

[39] Matava C.T., Kovatsis P.G., Lee J.K., Castro P., Denning S., Yu J., Park R., Lockman J.L., Von Ungern-Sternberg B., Sabato S. (2020). PeDI-Collaborative. Pediatric Airway Management in COVID-19 Patients: Consensus Guidelines From the Society for Pediatric Anesthesia’s Pediatric Difficult Intubation Collaborative and the Canadian Pediatric Anesthesia Society. Anesth. Analg..

[40] Hill C.S., Muirhead W.R., Vakharia V.N., Marcus H.J., Choi D. (2020). An Exit Strategy for Resuming Nonemergency Neurosurgery after Severe Acute Respiratory Syndrome Coronavirus 2: A United Kingdom Perspective. World Neurosurg..

[41] Munda M., Velnar T., Prestor B., Zele T., Spazzapan P., Matos B., Kos N., Benedicic M., Jeglic A., Tekavcic I. (2003). Neurosurgical service during COVID-19 pandemics in Ljubljana, Slovenia-lessons learned. Neurohirurgija.

